# The Fabrication of Alginate–Carboxymethyl Cellulose-Based Composites and Drug Release Profiles

**DOI:** 10.3390/polym14173604

**Published:** 2022-09-01

**Authors:** Svetlana Morozkina, Ulyana Strekalovskaya, Anna Vanina, Petr Snetkov, Alexander Krasichkov, Victoriya Polyakova, Mayya Uspenskaya

**Affiliations:** 1 Chemistry Engineering Centre, ITMO University, Kronverkskiy Prospekt, 49A, 197101 Saint Petersburg, Russia; 2 Departments of Radio Engineering Systems, Electrotechnical University “LETI”, Professor Popova Street 5F, 197022 Saint Petersburg, Russia; 3 Saint Petersburg Research Institute of Phthisiopulmonology, Ligovsky 2-4, 191036 Saint Petersburg, Russia

**Keywords:** polymer matrix, sodium alginate, carboxymethyl cellulose, crosslinking agent, films, hydrogels, drug release

## Abstract

Recently, hydrogels based on natural water-soluble polysaccharides have attracted more and more attention due to their favorable characteristics. The high water-holding capacity, lack of toxicity, and biodegradability of such hydrogels make it possible to develop new materials on their basis for biotechnological, biomedical, pharmacological, and medical purposes. Sodium alginate is a non-toxic natural polysaccharide found in marine algae. It is capable of forming solid gels under the action of polyvalent cations that cross-link polysaccharide chains. Alginate-based products are popular in many industries, including food processing, pharmaceutical, and biomedical applications. Cellulose is the most abundant, renewable, and natural polymer on Earth, and it is used for various industrial and biomedical applications. Carboxymethyl cellulose (CMC) is useful in pharmaceutical, food, and non-food industries such as tablets, ice cream, drinks, toothpaste, and detergents. In this review, various methods for the preparation of the compositions based on sodium alginate and CMC using different crosslinking agents have been collected for the first time. Additionally, the drug release profile from such polymer matrixes was analyzed.

## 1. Introduction

Natural polysaccharides and compositions based on such polymers are extremely valuable due to their various pharmacological characteristics. Natural polysaccharides (for example, alginates (Alg) and carboxymethyl cellulose (CMC) are becoming more and more popular due to their use in the obtaining of materials with a full-interpenetrating polymer network (IPN), which provides the improved mechanical properties, thermal stability, chemical resistance, and swelling capacity. Such materials possess biodegradability, good stability, non-cytotoxicity, biocompatibility, non-immunogenicity, and economical efficiency, which is why they can be used in engineering materials, biomedical applications, in the drug delivery systems, and as antiadhesive agents [1,2]. Besides full IPNs materials, it is possible to obtain materials based on semi-IPNs, sequential IPNs or sequential semi-IPNs: usually the properties of such materials are almost similar to IPNs material [3].

**Figure 1:**
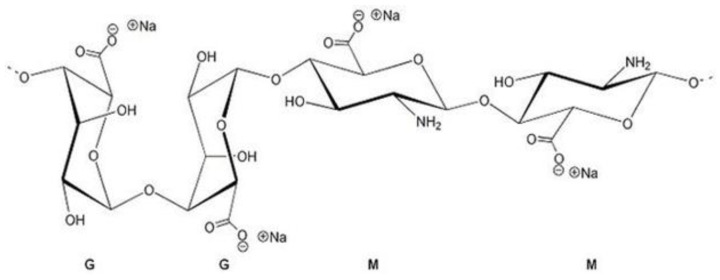
Sodium alginate structure.

Alginates represent linear anionic polysaccharide that includes blocks of β-(1-4)-D-mannuronic, called M-blocks and α-L-guluronic acid, called G-blocks [4]. The structure is shown in Figure 1. They are available from a variety of sources of seaweed. The brown seaweed’s cell wall contains a huge amount of alginic acid and sodium alginate, unlike other (red, green) macroalgae. In this condition, salt is insoluble. To improve the applicability of alginate, it can be separated from macroalgae as a water-soluble alginate using several methods [5].

The main method for the isolation of alginate from algae: algae is pretreated, alginate is extracted, then alginic acid is precipitated, after which alginic acid salts (sodium, potassium, calcium, etc.) may be obtained. The extraction of alginate can be realized using mineral acids with the pH about 1.5 [6]. The extraction efficiency of alginates depends on the pH. At low pH (acidic medium), the process is complicated due to the formation of insoluble alginic acid. At high pH (alkaline medium), the process is highly efficient [7]. Another method is ultrasonic extraction. Ultrasound provides the minimal use of destructive chemicals and increases the extraction by 4 times [8]. There is also the method of subcritical extraction with water, and it demonstrates great efficiency and high alginate yield [9].

The water-soluble sodium alginate is reported to have notable rheological effects such as gelation, thickening, and dispersion stabilization. These characteristics are related to the chemical structure of the alginate. Uronic acids can be structured in the form of heteropolymers (which contain blocks of mannuronic and guluronic acids) [10]. The proportion of mannuronic acid and guluronic acid M and G blocks and their structure significantly affect on the properties of alginate [11]. Alginate with a high content of M blocks can be applied for chronic wound healing due to its ability to generate cytokines through human white blood cells [12]. The quality of alginate mainly depends on the origin of the seaweed. In addition, alginate is a hydrophilic biopolymer that is harmless for people, biocompatible, biodegradable, non-genotoxic, and biostable. These outstanding features accelerate its usefulness in a variety of biomedical applications [13]. Alginate is the most widely used polymer for wound healing [5].

Cellulose is the most popular, widely used, renewable natural polymer, and it is used in numerous advanced clinical, industrial, and biomedical areas [14]. Cellulose is a linear polysaccharide of glucose which is an important component of cells of plants, bacteria, seaweed, and mushrooms. Industrial cellulose fibers are mainly obtained from wood, leaves, jute, bark, and cotton in the form of cellulose (natural fibers) or derived from natural cellulose (synthetic fibers) [15].

The main problem of cellulose for in vivo applications is its biodurability, connected with the absence of cellulases in animal and human tissues.

Biodegradability is the ability of the material to be degraded by microorganisms and bioresorbability is the ability to be digested or metabolized when implanted in vivo. The chemical modification and/or crosslinking of water-soluble cellulosics with bioresorbable components can result in the resorbable cellulose-based materials [16].

**Figure 2:**
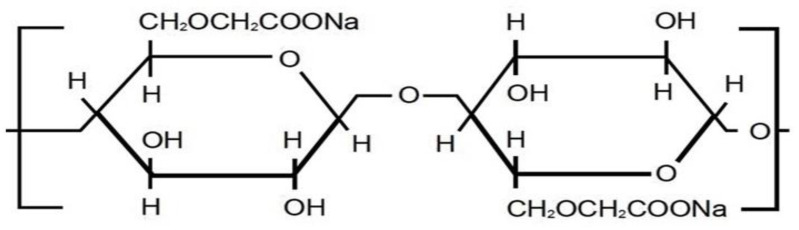
CMC structure.

Carboxymethyl cellulose (CMC) is cellulose ester, which has attracted commercial importance since the 1920s [17]. The structure is shown in Figure 2. CMC may be prepared by simple synthetic methods from widely available raw materials. CMC is used in such applications as in the paper, textile, pharmaceutical field, cosmetic, and food industries [18]. CMC is mostly favored over other cellulose derivations due to its gelling, emulsifying, binding, and thickening properties. These significant features provide the CMC use in pharmaceutics, food, and other food-related industries such as pills, ice cream, drinks, toothpaste, and detergents [19]. CMC can be used in combination with other polymers. This fact is very important for the development of the matrixes for drug encapsulation for biomaterials, hydrogels, and nanoparticles [20].

Crosslinking of alginate and CMC may be realized by the use of various cross-linking molecules in the dependence from the demands for the precise regulation of the crosslinking degree and the degree of the gel swelling, as well as for the preparation of the hydrogel with the controllable mechanical properties. The cross-linking agent is able to increase the tension strength and thermostability of films while decreasing their hydrophilicity [21]. Divalent cations, such as calcium ions, bind the alginate chains, resulting in the formation of network points with ionic cross-links, leading to the gelation of the alginate solutions [22]. These gels are commonly used in biomedical applications [23]. Sodium alginate (NaAlg) crosslinked with calcium ions with the formation of calcium alginate hydrogel, in which sodium ions are changed with calcium ions, and fragments of the molecular chain fit into the structure of an egg box with the formation of a crosslinking point [24]. The water absorption in calcium alginate hydrogel can reach up to 99%. At a low content of calcium ions, alginate forms the temporary crosslinking. Therefore, it exhibits high viscosity and thixotropy. With a high calcium content, permanent crosslinking is formed, and the tension strength increases. At the same time, the degree of swelling decreases. In practical applications, sodium alginate hydrogel crosslinked with calcium ions is usually obtained in nano size in the form of micelles or beads [25] because the hydrogel is very fragile, and the mechanical properties are poor. There are many hydroxyl groups in CMC, that, besides exhibition superb water absorption, can also bind with alginate molecules to form hydrogen bonds to increase the durability of the hydrogel [26].

**Figure 3:**
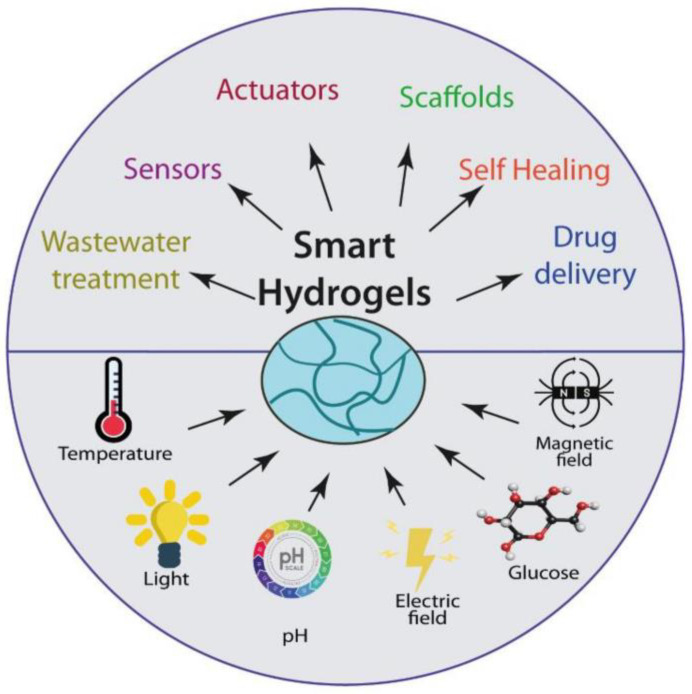
Stimulus-responsive hydrogels and their applications [27].

The main applications of hydrogels in the biomedicine field, like scaffolds for tissue engineering, actuators, drug delivery systems, biosensors, and many other applications, are reviewed [27] (Figure 3).

The theory describing the sorption thermodynamics of hydrogels, the relationships between their microstructural parameters, and the resulting macroscopic properties as well as various approaches to evaluate the most relevant network parameters affecting the hydrogel swelling capability and mechanical stiffness, are very well discussed in the review [28].

This review covers all known methods for the preparation of compositions based on sodium alginate and CMC—both the use of various crosslinking agents and the use of physical crosslinking of polymers. As a result, not only polymer films were obtained, but also other forms, such as hydrogel beads, nanofibers, films, nanoparticles, etc., may be developed. Furthermore, the majority of these compositions were used as the drug delivery systems, and biologically active substance release profiles were analyzed in this review.

## 2. The Preparation of Films and Beads Based on Alginate/Sodium Alginate and CMC

Alginate composites may be prepared by the ionic crosslinking with calcium, magnesium, barium, lead, cadmium, cobalt, zinc, nickel, manganese, strontium ions, etc., to form hydrogels [13,29,30,31,32].

Crosslinking agents such as Al^3+^, Ca^2+^, Mn2+, and Zn^2+^ are usually used to prepare alginate films with the improved mechanical properties [33].

There is limitation for the use of the crosslinked alginate in practical use due to the effect of acidic medium on the crosslinking of the film and as the result on the drug release rate [34].

It is known that cellulose, alginate, and its derivatives may form a stable network via the crosslinking [35,36].

The mixing of CMC with alginate results in high strength and higher tensile strength and elongation at break [37,38].

Alginate/CMC (3:1 ratio) films were prepared by the crosslinking in 2% *w*/*v* of the ionic solutions (BaCl_2_—pH = 5.23; CaCl—pH = 5.27 and ZnCl_2_—pH = 5.28) for 1 min [39].

Alginate/CMC films were also obtained by the crosslinking with glutaraldehyde and copper sulfate. The solutions with the various ratios of alginate to CMC (0:10, 2:8, 4:6, 6:4, 8:2, and 10:0) in distilled water at 60 °C for 24 h were prepared and cross-linked by the addition of glutaraldehyde (25% *w*/*v*) at 0, 2, 5%, and/or copper sulfate of 0 and 0.02 under stirring for 30 min. Then, the homogeneous mixtures were cast into the Petri dishes, and dried at 40 °C. The obtained hydrogels have better swelling time. The increase in alginate ratio and Cu^2+^ content results in an increase of the percentages of solid that remains [40].

Monolayer and bilayer films as wound dressing materials were obtained with Alg/CMC ratios (weight percentages) of 0:100, 25:75, 50:50, 75:25, and 100:0. Homogeneous, thin, and continuous films were obtained using glycerol and crosslinking with CaCl_2_. The film characteristics have the next tendency: intermediary characteristics from both polymers have been observed for the polymer ratio 50:50; the increase of CMC leads to the improvement of the liquid behavior characteristics, while the mechanical properties worsened. The bilayer film (50:50) has better water vapor transmission rate in comparison with the monolayer film (50:50) [41].

The incorporation of alginate and CMC into pullulan results in the significant weakness of water barrier and mechanical properties. The film solubilization time in water was reduced when pullulan was blended with alginate or CMC up to about 17–33% (*w*/*w* total polymer). The addition of glycerol further leads to the reduction of tensile strength, increased elongation at break, weakened water barrier properties, and enhanced solubilization in water. The blending pullulan with alginate and CMC results in weaker hydrogen bonds acting on –OH groups compared to the pure pullulan [42].

The CMC/carbon dots films were prepared as described in the publication [43]. For this, carbon dots with a higher quantum yield from the experimental Box–Behnken scheme were used. The solution of carbon dots was added after the dissolving of CMC and NaAlg in distilled water with glycerol. After homogenization and gel formation, it was centrifuged. Finally, the mixture was poured out onto a glass plate and stored for 24 h at 40 °C [44]. The resulting materials had enhanced thermal and mechanical stability.

Cu^2+^ ions and Ca^2+^ ions were used as cross-linkers to obtain the capsules with pure alginate and alginate with CMC (5 wt% and 10 wt%) and significant influence of the type of the crosslinking agent on physicochemical properties of the composites, as well as the binding and release of Cu^2+^ ions, was observed. Three aqueous solutions were used: pH 3, pH 7, and 1 wt% NaNO_3_. Desorption was carried out for 14 days. Kinetics of enrichment with Cu^2+^ ions of composites crosslinked with Ca^2+^ ions has been studied [45].

Synthesis of NaAlg–CMC beads with larger specific surface area and porosity due to the internal network structure through the cross-linking in CaCl_2_ and FeCl_3_ solutions were performed. The Pb(II) adsorption of the beads followed the Langmuir adsorption isotherm and exceeded 99%, and the lead ion removal efficiency was significantly higher than that of conventional adsorbents. The pseudo-second-order rate equation describes the dynamic adsorption model [46].

Methylenebisacrylamide (MBA) as the crosslinking agent and ammonium persulfate (APS) as an initiator were used for the preparation of superabsorbent hydrogel based on CMC and NaAlg. The swelling kinetics of the hydrogels depend on particle size, MBA concentration, and pH. The water absorbency for the hydrogels in monovalent cations salt solutions is in the order LiCl > NaCl > KCl, and in the order Na^+^ > Ca^2+^ > Al^3+^ for NaCl, CaCl_2_, and AlCl_3_ aqueous salt solutions, while the increase of MBA and APS concentration results in water absorbance decrease [47].

The compatibility between CMC and NaAlg (20, 30, and 40%) was confirmed when the gel was obtained with irradiation dose up to 20 kGy of gamma rays. The increasing of CMC content and irradiation dose up to 20 kGy results in the improved mechanical and thermal properties. The good antimicrobial activity against Gram +ve Bacteria (*Bacillus subtilis*) was confirmed [48].

Meticillin-resistant *Staphylococcus aureus*, Meticillin-resistant *Staphylococcus epidermidis*, Vancomycin-resistant *Enterococcus faecalis*, *Streptococcus pyogenes*, *Staphylococcus epidermidis*, *Escherichia coli*, *Pseudomonas aeruginosa*, and *Candida albicans* were used to evaluate the activity and efficacy of alginate/CMC dressing, developed for the treatment of acute and chronic wounds, specifically burns. The obtained dressing demonstrates antimicrobial activity against all eight microorganisms in the 21-day study, which has an advantage over the poor efficacy of the commercial Aquacel Ag against *Staphylococcus epidermidis*, *Escherichia coli*, *Candida albicans*, and *Pseudomonas aeuginosa* after 14 days [49].

## 3. The Drug-Loaded Alginate/Sodium Alginate—CMC Based Polymer Matrixes

For the development of edible films, having good carrying capacity, required barrier properties, ecological safety, and antibacterial protection of foods, NaAlg was used as the film-forming polymer. NaCMC plays role of modifier, and glycerol was used as a plasticizer. For bacteriostatic properties, *Lactococcus lactis* (1.5 g/100 g) were added to the film solutions, resulting in the decrease of transparency and a significant increase in red and yellow tints, while thickness was not changed significantly. The tensile strength of the films was slightly decreased, and water vapor permeability was significantly higher. The encapsulation efficiency of BSA-FITC by AL2 beads was 96.12 ± 2.21%, slightly lower than 87.76 ± 3.85%, observed in beads with a higher volume ratio of CMC2. The films possess significant bacteriostatic activity against *Staphylococcus aureus* at 4 °C [50].

To develop the potential drug delivery systems for mucosal surfaces including wounds, the NaAlg-CMC-based freeze-dried wafers and CMC-based films were prepared. Higher paracetamol loading and water absorption capacity was detected in the case of beads than the corresponding solvent evaporated films. The polymer and drug concentration influence moisture absorption, ease of hydration, and mechanical behaviour [51].

Sodium alginate and CMC hydrogels can be used for the encapsulation and controlled release of furazolidone and bismuth(III) [52]. Sodium alginate and CMC were dissolved in sodium hydroxide solution and stirred under different modes and temperatures for 24 h. Next, diethanolamine was added, and the resulting mixture was stirred for 30 min at 60 °C. The films were prepared by pouring the solution into the plate, avoiding the formation of bubbles, and were dried in an oven at 40 °C. The crosslinking of the resulting films was conducted by soaking them for 15 min in the aqueous solution of calcium chloride (2% *w*/*v*). The research showed that the properties of the obtained hydrogel films, for instance, the swelling rate and release of active components, depend on the pH of the medium. Thus, the films with Bi^3+^ were sustainable and showed a good drug release profile in neutral solutions. The expected antimicrobial activity of films with Bi^3+^ and furazolidone was confirmed.

Films with diclofenac were prepared by two stages of crosslinking [53]. The typical procedure is described as follows. NaAlg and CMC were separately dissolved in aqueous solutions containing 3% *w*/*v* glycerol. 1% *w*/*v* calcium chloride was added to the NaAlg film solution to perform the first crosslinking step. Both solutions (NaAlg and CMC in glycerol) were stirred at 900 rpm and heating at 50 °C. After the deaeration in vacuum, aliquots of the dispersions mixed with NaAlg and CMC were poured into Petri dishes and dried for 20 h at 40 °C in a convection oven. For two-layer films, a film-like solution was prepared, half of which was poured into Petri dishes, and the rest of which was added to the material after 10 h. The resulting films were immersed for 20 min in the aqueous solution of calcium chloride and glycerol for the second stage of the crosslinking. The films with diclofenac have lower mechanical properties and physical properties similar to the films without the drug. The influence of drug molecules on the surface properties and on the proliferation of keratinocytes and skin fibroblasts have been evaluated.

Multilayer thin films on the silicon wafers were obtained in the study [54]. The pieces of silicon wafers were saturated with a solution of hydrogen peroxide and sulfuric acid (conc) and then washed with pure water and dried under the flow of dry nitrogen. To obtain films with several layers, the mixture of NaAlg and CMC was placed onto the prepared silicon wafers, placed in a centrifuge, and rotated. The obtained nanoscale thin films showed significant encapsulation efficiency and ‘burst’ drug release profile for anesthetics such as diclofenac and lidocaine.

Diclofenac was loaded into the gel based on Na-CMC, obtained under the action of AlCl_3_ as a cross-linker. The release rate of diclofenac was significantly reduced when the beads was coated with NaAlg to increase the pathway of the diffused medium and increased with AlCl_3_ concentration increasing. The higher AlCl_3_ concentrations (40 and 60% *w*/*v*) result in more uniform and rounded beads than in the case of 20% *w*/*v* salt. The particle size of the core beads decreased insignificantly as the flocculating agent concentration increased [55].

To evaluate the ophthalmic mucoadhesive system and its in vitro antibacterial potential on pathogenic microorganisms, *Staphylococcus aureus* and *Escherichia coli*, the gatifloxacin was encapsulated into the NaAlg and NaCMC-based polymer system. The enhancement of the gel bioadhesion properties was achieved due to NaCMC presence. The mucoadhesive force was enhanced significantly when the concentration of NaAlg and NaCMC were increased. The properties and concentration of NaAlg and NaCMC significantly influence the in vitro release of gatifloxacin in simulated tear fluid (STF, pH—7.4). The gatifloxacin-loaded polymer system demonstrates a significant reduction in total bacterial count [56].

**Figure 4:**
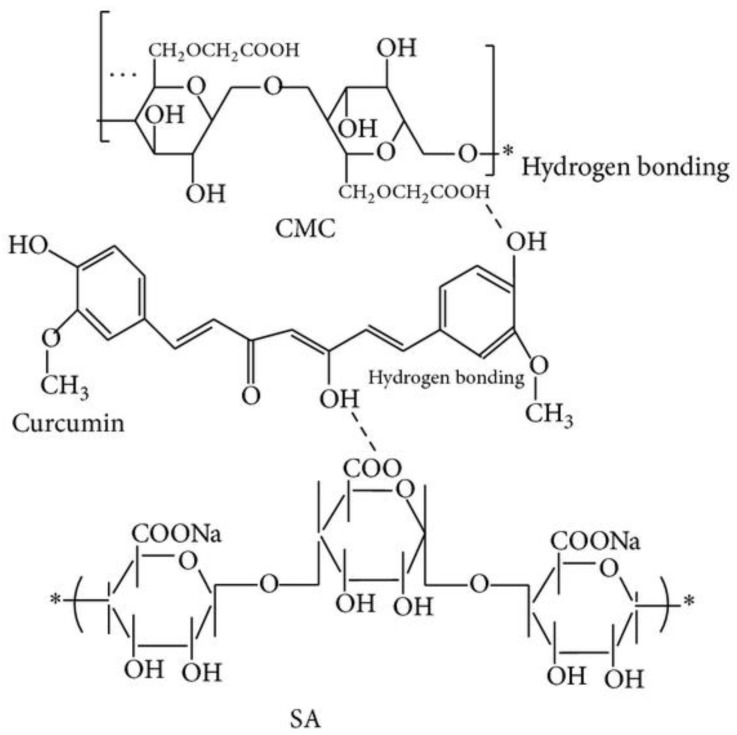
The structure of curcumin–alginate–CMC beads [57].

The loading of turmeric extract into the polymer matrix prepared by the cross-linking with CaCl_2_ allowed us to obtain the smallest particles when sodium alginate and CMC were blended in the ratio of 10:5, with the formation of the hydrogen interaction between hydroxyl group of curcumin with carboxylic group in the polymers (Figure 4). The increase of CMC concentration leads to the increasing of the negative zeta-potential, while the increase of CaCl_2_ decreases the swelling ratio.

Turmeric extract concentration influences spherical, egg, and rectangular shape formation. Higher release rates were demonstrated in the case of higher extract concentrations. The polymer beads without turmeric extract have 80% of growth inhibition of human colon adenocarcinoma cells, which may be explained by the interaction of polymeric carboxylic group with colon cancer cells. The beads with the concentration of the turmeric extract from 1.0 to 2.5% possess the growth inhibition of colon cancer cells comparable to 2.5% pure turmeric extract (97–98%) [57].

It was observed that the roughness and pore size of alginate–CMC beads were obtained by the dropping of the solution into FeCl_3_, as the cross-linker was increased with the CMC volume ratio. A three-dimensional structure was detected by FTIR analysis. The simulated gastrointestinal conditions (pH 1.2, 4.5, and 7.4) were used for the evaluation of the swelling degree and the albumin release profile, which depend on volume ratios of the polymer matrix and pH [58].

IPN beads based on high molecular weight alginate (220,000 g/mol) and CMC were fabricated via ionic the crosslinking with AlCl_3_, FeCl_3_, CrCl_3_, and CaCl_2_. The solution was extruded out and the formed droplets were immediately placed into a bath with 0.1 M cross-linker solution for 1 h, and then the beads were placed in deionized water. The sigmoidal pattern, with the initial delay before more extensive release, was observed when Bovine serum albumin (2 mg/mL) was encapsulated into the polymer matrix. The viability and green fluorescent protein (GFP+) expression from the GFP+ *E. coli* were also evaluated [59].

The beads on the base of sodium alginate and CMC were prepared using BaCl_2_ as a cross-linker [60]. For this purpose, the polymers were dissolved in water at 40 °C and stirred for 1 h. Next, the hydrogel beads were prepared by the solution extruding through a syringe. The polymer was dropped into the gel-forming solution—20% *w*/*v* barium chloride—and stirred. Next, the beads were filtered and placed in deionized water for 1 min in order to clean the surface from the excess of BaCl_2_. The resulting beads were dried for 24 h at the room temperature. It was demonstrated that the resulting beads are capable of incorporating the drug methotrexate and have a good drug release profile. The drug was released completely over 5 h.

pH sensitive hydrogel beads based on NaAlg/NaCMC blend were prepared by the crosslinking in the ferric chloride solution as drug delivery system for Metformin hydrochloride (MH). The encapsulation efficiency, swelling, and in vitro release profiles of the beads strongly depended on FeCl_3_ solution; beads have spherical surface, and a dramatic reduction of size when FeCl_3_ concentration was increased. The beads at pH 7.4 demonstrated higher swelling properties compare to pH 1.2 in gastric and intestine stimuli atmosphere at 37 °C. The higher release profiles were at pH 7.4 compared to pH 1.2 [61].

Alzheimer’s drug, donepezil hydrochloride (DP), was loaded into microspheres of poly(vinyl alcohol)-grafted polyacrylamide (PVA-g-PAAm)/sodium alginate (NaAlg)/NaCMC, prepared by the emulsion-crosslinking method using FeCl_3_, CaCl_2_, AlCl_3_, and ZnCl_2_. A higher percentage of entrapment efficiency compared to the microspheres prepared with other crosslinkers was obtained when FeCl_3_ was used. The release of DP (in gastric (2 h)–pH = 1.2, input intestinal (2 h)–pH = 6.8, and intestinal (2 h)–pH = 1.2 at 37 °C) increased with the increase in drug/polymer ratio (d/p) and PVA-g-PAAm/NaAlg/NaCMC ratio, while it decreased with the increase in the extent of crosslinking. The optimum DP release was 92.9% for a PVA-g-PAAm/NaAlg/NaCMC with the ratio as 1/2/1, d/p ratio as 1/8, and FeCl_3_ concentration of 7% (*w*/*v*) [62].

Nitrogen-phosphorus-potassium (NPK) fertilizers in the form of NH_4_Cl and KH_2_PO_4_ were introduced into the stable dispersions of NaAlg and CMC (3.0% (*wt*/*vol*), obtained with citric acid and CaCl_2_ as the cross-linkers. The polymer ratios were 1:2, 1:1, and 2:1 at the constant crosslinker amount of 5% (wt/wt polymer), and the amount of crosslinker was 5% and 10% (*wt*/*wt* polymer) at the constant polymer volume ratio (1:1). The formed particles have spherical shape with the size from 733 to 1200 nm, which depend on polymer ratios and cross-linker concentration. The highest encapsulation efficiency (86–91%) was achieved in the case of the 1:1 CMC/Alg ratio with 10% citric acid (per weight polymer). The maximum release rate (50%) of NPK from polymer matrix was in 30 days. The Korsmeyer–Peppas mathematical mode describes the release mechanism, demonstrating that the release behavior is both governed by polymer relaxation and diffusion [63].

## 4. Release Profile of Biologically Active Substances from Alginate/CMC Polymer Matrixes

The drug release profile of biologically active substances (BASs) from different polymer compositions is shown in Table 1. Release profile depended on drug nature, structure, and nature of polymer matrix.

## 5. Commercially Available Pharmaceutical Alginate–CMC Based Products

Despite the fact that the commercially available alginate–CMC-based products have been already highlighted in the review [64], we discussed their use in this review to provide full current information about alginate–CMC based products (Table 2).

Silvercel™ is a non-woven pad composed of silver coated nylon fibers, high G (guluronic acid) alginate, and CMC. It is proposed as a suitable dressing for the management of chronic wounds with broad-spectrum antimicrobial action, high absorbency, high wet tensile strength (increased by 56% when wet compared to dry based on in vitro data), absorbing exudate in moderate to heavily exuding wounds (pressure ulcers, venous leg ulcers, diabetic foot ulcers, donor sites, traumatic and surgical wounds, partial-thickness wounds). The dressing that is in contact with the wound may remain on the surface of the wound over an extended period of time, and in some cases, until the wound is completely healed.

Comfeel Plus™ can be used for the treatment of low and moderate exudate of wounds during wound healing, and it is used for pressure ulcers, leg ulcers, superficial burns, donor sites, post-operative wounds, and skin abrasions. The dressing can stay on for up to 7 days [72].

## 6. Conclusions and Future Perspectives

The use of bioavailable, safe, biodegradable polymers with various properties is becoming more widespread. The combination of sodium alginate and CMC is used for the preparation of various formulations. One of the areas of the development is the creation of wound-healing compositions based on them, with a biologically active substance in the polymer matrix. Various compositions were prepared, with crosslinking agents used, and the resulting composites formed. The main preparation products were films, sponges, and nanoparticles. Films based on alginate and CMC may be obtained using calcium chloride, sodium hydroxide/calcium chloride, 0.1 wt% N,N-methylenebis-acrylamide, multilayer thin films obtained using Silicon wafers, gel/films obtained using carbon dots, and beads, in the case of barium chloride as cross-linker.

The most commonly used crosslinking agent is calcium chloride due to its non-toxicity and accessibility, unlike other types of crosslinkers.

It is possible to offer many options for the creation of such complexes by the changing of the preparation parameters: different ratios of the polymers themselves, and the use of various drugs.

One of the promising directions may be the development of enzyme-containing hydrogels [74], and another one is vaccine delivery [75].

## Figures and Tables

**Table 1:** Release profile of a biologically active substance from polymer matrixes.

No.	Biologically Active Substance/Polymer Matrix	Release Profile	Ref.
1	*Lactococcus lactis*/NaAlg-NaCMC Films	The BSA-FITC release from AL2 beads was slow, 3.46 ± 1.33% and 7.41 ± 0.85% of the total encapsulated BSAFITC. The r samples A2C1, A1C1, and A1C2 showed similar BSA-FITC release behavior, which may be explained by the carboxyl groups of the alginate being protonated at pH 1.2, and a low degree of swelling limited diffusion of BSAFITC. The release rate of BSA-FITC from AL2 beads was faster than from A2C1, A1C1, and A1C2 beads due to the higher degree of swelling at pH 7.4. The A1C2 beads provided the controlled release from A1C2 beads over longer periods of time in the intestines.	[50]
2	Furazolidone and Bismuth (III)/NaAlg–CMC hydrogel	In a neutral solution, SCFDZ releases almost all the entrapped drug in about 8 h, the SCFDZ-Bi formulation releases around 60% of the encapsulated FDZ in the same period and continues releasing the drug for more than 24 h. At pH 1.2, the effect of bismuth(III) ions on the release rate of FDZ was less significant.	[52]
3	Diclofenac/Alg-CMC films	Released over a period of 420 min for the MLD film and 600 min for the BLD film. The MLD film showed a burst release up to 60 min with 59%, differently for the BLD films, when only 26% was released over this same time. After 360 min of contact between the films and the liquid media, the release of diclofenac reached values of 0.147 ± 0.021 mg cm^−2^ for the MLD film and 0.166 ± 0.016 mg cm^−2^ for the BLD film. No statistically significant difference between these films was observed, and the amount of diclofenac released was higher than the recommended dosage.	[53]
4	diclofenac and lidocaine/NaAlg/CMC	The fast (burst) release (∼45%) within the first 30 min may be explained by the dissolution of “trapped” or unbound drug molecules within the polymer structure. The latter is followed by a slower and prolonged release (∼50%) for the next 6 h (360 min), which is related to the combination of swelling and partial erosion. The Alg-CMC has the most sustained release. The overall released mass of DCF and LID from three types of multi-layers are compared. The release properties of both drugs are suited for Korsmeyer–Peppas model. The release behavior is predicted to be non-Fickian based on the exponent “n” value, which is above 0.89 in all cases.	[54]
5	diclofenac sodium/NaAlg/AlCMC	There is a linear relationship for drug release over a 4–5 h period from the core beads where it showed a longer straight line relationship (7 h) for the coated beads. The coating of the Al-CMC beads, prepared using 60% *w*/*v* AlCl_3_ × 6H_2_O, with 2% *w*/*v* aqueous solution of NaAlg resulted in a significant sustaining action of the Al-CMC beads as indicated by the shift in T_max_ from 1.7 ± 0.84 to 3 ± 0.71 h and the prolongation of the MRT from 7.86 ± 0.54 to 10.82 ± 1.33 h. The C_max_ increased significantly from 5.43 ± 2.91 to 11.66 ± 6.18 μg/mL, while the AUC0 → 24 increased significantly from 39.82 ± 26.61 to 57.92 ± 25.58 μg h/mL, resulting in a relative bioavailability of 145.45% relative to Al-CMC beads.	[55]
6	Gatifloxacin/NaAlg/NaCMC	Comparison of the release profile of GS2 (containing only NaAlg) with those of GS3–GS5 indicate that burst effect was considerably reduced. NaCMC (incorporated at 0.1 to 0.5% *w*/*v*) affected the drug release significantly.	[56]
7	Turmeric Extract/NaAlg/CMC	Higher tmr contents clearly results in higher release rates. The *n* value increased with increasing of tmr content. The release pattern was not Fickian diffusion controlled. The dependence of the *n*-value on tmr concentration was found.	[57]
8	Albumin/Alg/CMC	The release profile of albumin from the beads were investigated under simulated gastrointestinal conditions (pH 1.2, 4.5, and 7.4). The Fe^3+^– crosslinked beads displayed different degrees of albumin release for the various volume ratios and under various pH conditions. The Fe^3+^–crosslinked AC beads protected and controlled the release of protein.	[58]
9	Methotrexate/NaAlg/NaCMC	Almost the total amount of MTX release (98.1 ± 2.64%) was completed in 5 h. Entrapment efficiency (EE%) and release behaviors of the metal complexes of MTX were compared to MTX. The mostly used drug release kinetic models, which are first order, Higuchi, and Hixson-Crowell, were applied to release data.	[60]
10	Metformin hydrochloride/NaAlg/NaCMC	The higher release profiles were pH 7.4 compared to pH 1.2.	[61]
11	poly(vinyl alcohol)-grafted polyacrylamide (PVA-g-PAAm)/NaAlg/NaCMC	The release was studied in 250 mL of HCl (pH 1.2) and PBS (pH 6.8 and 7.4).The release of DP increased with the increase in drug/polymer ratio (d/p) and PVA-g-PAAm/NaAlg/NaCMC ratio, while it decreased with the increase in the extent of crosslinking. The optimum DP release was 92.9% for a PVA-g-PAAm/NaAlg/NaCMC with the ratio as 1/2/1, d/p ratio as 1/8, and FeCl_3_ concentration of 7% (*w*/*v*).	[62]

**Table 2:** Commercially available alginate–CMC products and their effects.

Name	Composition/Target	Study Type/Effects	Ref.
Aquacel™ Ag EXTRA™ Hydrofiber™ (Prescribed/OTC)	Bi-layer Na Alg CMC—Ag+—strengthening fibers; Surgical, traumatic, exuding, infected, painful wounds, second degree burns, ulcers, or for minor cuts or burns	Bacterial inhibition, absorbs the wound exudate while forming a gel, it must be changed every 1–2 weeks	[65]
Purilon Gel^®^ (ColoPlast)	Na CMC—Ca Alg; Used with another dressing for first and second degree burns or sloughy and necrotic wounds	Wound surface moisturizer	[66,67]
Silvercel™ (Johnson & Johnson)	36% Ca ALG with high G—6% CMC—28% Ag (111 mg Ag/100 cm^2^)—30% EasyLift Precision Film (Acelity/Systagenix)	Pig and human trials, wound healing	[68,69,70,71,72]
Comfeel Plus™	NaCMC and calcium alginate	Ulcers such as venous leg ulcers, pressure ulcers; burns, donor sites, postoperative wounds, and necrotic wounds	[73]

## Data Availability

The data presented in this study are available on request from the corresponding author.

## References

[1] Shaikh M.A.J., Gilhotra R. (2021). Current update on psyllium and alginate incorporate for interpenetrating polymer network (IPN) and their biomedical applications. Int. J. Biol. Macromol..

[2] James J., Thomas G.V., Akhina H., Thomas S. (2016). Micro-and Nano-Structured Interpenetrating Polymer Networks: State of the Art, New Challenges and Opportunities. Micro-And Nano-Structured Interpenetrating Polymer Networks: From Design to Applications.

[3] Zoratto N., Matricardi P. (2018). Semi-IPNs and IPN-based hydrogels. Polym. Gels.

[4] Coleman R.J., Lawrie G. (2011). Phosphorylation of alginate: Synthesis, characterization, and evaluation of in vitro mineralization capacity. Biomacromolecules.

[5] Varaprasad K., Jayaramudu T. (2020). Alginate-based composite materials for wound dressing application: A mini review. Carbohydr. Polym..

[6] Faidi A., Lassoued M.A., Becheikh M.E.H., Touati M., Stumbé J.F., Farhat F. (2019). Application of sodium alginate extracted from a Tunisian brown algae Padina pavonica for essential oil encapsulation: Microspheres preparation, characterization and in vitro release study. Int. J. Biol. Mol..

[7] Charoensiddhi S., Conlon M.A., Vuaran M.S., Franco C.M., Zhang W. (2016). Impact of extraction processes on prebiotic potential of the brown seaweed Ecklonia radiata by in vitro human gut bacteria fermentation. J. Funct. Foods.

[8] Kartik A., Akhil D., Lakshmi D., Gopinath K.P., Arun J., Sivaramakrishnan R., Pugazhendhi A. (2021). A critical review on production of biopolymers from algae biomass and their applications. Bioresour. Technol..

[9] Gereniu C.R.N., Saravana P.S., Chun B.S. (2018). Recovery of carrageenan from Solomon Islands red seaweed using ionic liquid-assisted subcritical water extraction. Sep. Purif. Technol..

[10] Prasad C.V., Swamy B.Y. (2012). Formulation and characterization of sodium alginate g-hydroxy ethylacrylate bio-degradable polymeric beads: In vitro release studies. J. Polym. Environ..

[11] Varaprasad K., Raghavendra G.M. (2016). Nano zinc oxide–sodium alginate antibacterial cellulose fibres. Carbohydr. Polym..

[12] Del Gaudio P., Amante C. (2020). In situ gelling alginate-pectin blend particles loaded with Ac2-26: A new weapon to improve wound care armamentarium. Carbohydr. Polym..

[13] Lee K.Y., Mooney D.J. (2012). Alginate: Properties and biomedical applications. Prog. Polym. Sci..

[14] Yang J., Li J. (2018). Self-assembled cellulose materials for biomedicine: A review. Carbohydr. Polym..

[15] Klemm D., Heublein B. (2005). Cellulose: Fascinating biopolymer and sustainable raw material. Angew. Chem. Int. Ed..

[16] Sannino A., Demitri C., Madaghiele M. (2009). Biodegradable Cellulose-based Hydrogels: Design and Applications. Materials.

[17] Zennifer A., Senthilvelan P. (2020). Key advances of carboxymethyl cellulose in tissue engineering & 3D bioprinting applications. Carbohydr. Polym..

[18] Agarwal R., Ko K.R. (2017). Biopatterning of keratinocytes in aqueous two-phase systems as a potential tool for skin tissue engineering. MRS Adv..

[19] Thakur V.K. (2015). Cellulose-Based Graft Copolymers: Structure and Chemistry.

[20] Mejía E.H., Contreras H. (2019). Effect of Experimental Parameters on the Formation of Hydrogels by Polyelectrolyte Complexation of Carboxymethylcellulose, Carboxymethyl Starch, and Alginic Acid with Chitosan. Int. J. Chem. Eng..

[21] Aljawish A., Munigalia L. (2016). Characterization of films based on enzymatically modified chitosan derivatives with phenol compounds. Food Hydrocoll..

[22] George M., Abraham T.E. (2007). pH sensitive alginate–guar gum hydrogel for the controlled delivery of protein drugs. Int. J. Pharm..

[23] Pankongadisak P., Ruktanonchai U.R. (2015). Development of silver nanoparticles-loaded calcium alginate beads embedded in gelatin scaffolds for use as wound dressings. Polym. Int..

[24] Hecht H., Srebnik S. (2016). Structural characterization of sodium alginate and calcium alginate. Biomacromolecules.

[25] Wang X.F., Lu P.-J. (2016). Nano hydroxyapatite particles promote osteogenesis in a three-dimensional bio-printing construct consisting of alginate/gelatin/hASCs. RSC Adv..

[26] He X., Zeng L. (2021). Shape memory composite hydrogel based on sodium alginate dual crosslinked network with carboxymethyl cellulose. Eur. Polym. J..

[27] Bustamante-Torres M., Romero-Fierro D., Arcentales-Vera B., Palomino K., Magaña H., Bucio E. (2021). Hydrogels Classification According to the Physical or Chemical Interactions and as Stimuli-Sensitive Materials. Gels.

[28] Sannino A., Madaghiele M. (2009). Biocompatibility and other properties of hydrogels in regenerative medicine. Cellular Response to Biomaterials.

[29] Aderibigbe B.A., Buyana B. (2018). Alginate in Wound Dressings. Pharmaceutics.

[30] Topuz F., Henke A., Richtering W., Groll J. (2012). Magnesium ions and alginate do form hydrogels: A rheological study. Soft Matter.

[31] Yin M., Xu F., Ding H., Tan F., Song F., Wang J. (2015). Incorporation of magnesium ions into photo-crosslinked alginate hydrogel enhanced cell adhesion ability. J. Tissue Eng. Regen. Med..

[32] Olukman M., Şanlı O., Solak E. (2012). Release of Anticancer Drug 5-Fluorouracil from Different Ionically Crosslinked Alginate Beads. J. Biomater. Nanobiotechnol..

[33] Liling G., Di Z., Jiachao X., Xin G., Xiaoting F., Qing Z. (2016). Effects of ionic crosslinking on physical and mechanical properties of alginate mulching films. Carbohydr. Polym..

[34] Al-Musa S., Abu Farab D., Badwana A. (1999). Evaluation of parameters involved in preparation and release of drug loaded in crosslinked matrices of alginate. J. Control. Release.

[35] Anirudhan T.S., Nima J., Divya P.L. (2015). Synthesis, characterization and in vitro cytotoxicity analysis of a novel cellulose based drug carrier for the controlled delivery of 5-fluorouracil, an anticancer drug. Appl. Surf. Sci..

[36] Demitri C., Del Sole R., Scalera F., Sannino A., Vasapollo G., Maffezzoli A., Ambrosio L., Nicolais L. (2008). Novel superabsorbent cellulose-based hydrogels crosslinked with citric acid. J. Appl. Polym. Sci..

[37] Han Y., Yu M., Wang L. (2018). Physical and antimicrobial properties of sodium alginate/carboxymethyl cellulose films incorporated with cinnamon essential oil. Food Packag. Shelf Life..

[38] Zheng J., Zeng R., Zhang F., Kan J. (2019). Effects of sodium carboxymethyl cellulose on rheological properties and gelation behaviors of sodium alginate induced by calcium ions. LWT Food Sci. Technol..

[39] Pinpru N., Woramongolchai S. (2020). Crosslinking Effects on Alginate/Carboxymethyl Cellulose Packaging Film Properties. Chiang Mai J. Sci..

[40] Sritweesinsub W., Charuchinda S. (2015). Alginate/Carboxymethyl Cellulose Hydrogel Films in Relation to Crosslinking with Glutaraldehyde and Copper Sulfate. MATEC Web Conf..

[41] Trevisol T.C., Fritz A.R.M., de Souza S.M.A.G.U., Bierhalz A.C.K., Valle J.A.B. (2018). Alginate and carboxymethyl cellulose in monolayer and bilayer films as wound dressings: Effect of the polymer ratio. J. Appl. Polym. Sci..

[42] Tong Q., Xiao Q., Lim L.-T. (2008). Preparation and properties of pullulan–alginate–carboxymethylcellulose blend films. Food Res. Int.

[43] You Y., Zhang H. (2016). Transparent sunlight conversion film based on carboxymethyl cellulose and carbon dots. Carbohydr. Polym..

[44] Da Silva Júnior A.H., Macuvele D.L.P. (2021). Polymeric Blends of Carboxymethyl Cellulose and Sodium Alginate Containing Functionalized Carbon Dots Result in Stable and Efficient Fluorescent Films for Silver and Iron (III) Sensing. J. Environ. Chem. Eng..

[45] Mikula K., ·Skrzypczak D., Ligas B., ·Witek-Krowiak A. (2019). Preparation of hydrogel composites using Ca^2+^ and Cu^2+^ ions as crosslinking agents. SN Appl. Sci..

[46] Ren H., Gao Z., Wu D., Jiang J., Sun Y., Luo C. (2016). Efficient Pb(II) removal using sodium alginate-carboxymethyl cellulose gel beads: Preparation, characterization, and adsorption mechanism. Carbohydr. Polym..

[47] Pourjavadi A., Barzegar S., Mahdavinia G.R. (2006). MBA crosslinked Na-Alg/CMC as a smart full-polysaccharide superabsorbent hydrogels. Carbohydr Polym..

[48] Ibrahim S.M., El Salmawi K.M. (2012). Preparation and properties of Carboxymethyl Cellulose (CMC)/Sodium alginate (SA) blends induced by gamma irradiation. J. Polym. Environ..

[49] Bradford C., Freeman R., Percival S.L. (2009). In Vitro Study of Sustained Antimicrobial Activity of a New Silver Alginate Dressing. J. Am. Col. Certif. Wound. Spec..

[50] Ye J., Ma D., Qin W., Liu W. (2018). Physical and Antibacterial Properties of Sodium Alginate—Sodium Carboxymethylcellulose Films Containing Lactococcus lactis. Molecules.

[51] Boateng J.S., Auffret A.D., Matthews K.H., Humphrey M.J., Stevens H.N.E., Eccleston G.M. (2010). Characterisation of freeze-dried wafers and solvent evaporated films as potential drug delivery systems to mucosal surfaces. Int. J. Pharm..

[52] Silva K.M.M.N., de Carvalho D.E.L. (2019). Concomitant and controlled release of furazolidone and bismuth (III) incorporated in a cross-linked sodium alginate-carboxymethyl cellulose hydrogel. Int. J. Biol. Macromol..

[53] Trevisol T.C., Scartazzini L. (2020). Diclofenac release from alginate/carboxymethyl cellulose mono and bilayer films for wound dressing applications. Cellulose.

[54] Maver T., Mohan T. (2019). Polysaccharide thin solid films for analgesic drug delivery and growth of human skin cells. Front. Chem..

[55] Hosny E.A., Al-Helw A.A.-R.M. (1998). Effect of coating of aluminum carboxymethylcellulose beads on the release and bioavailability of diclofenac sodium. Pharm. Acta Helv..

[56] Kesavan K. (2010). Sodium alginate based mucoadhesive system for Gatifloxacin and its in vitro antibacterial activity. Sci. Pharm..

[57] Riyajan S.A., Nuim J. (2013). Interaction of green polymer blend of modified sodium alginate and carboxylmethyl cellulose encapsulation of turmeric extract. Int. J. Polym. Sci..

[58] Kim M.S., Park S.J., Gu B.K., Kim C.H. (2012). Ionically crosslinked alginate-carboxymethyl cellulose beads for the delivery of protein therapeutics. Appl. Surf. Sci..

[59] Lee K., Hong J., Roh H.J., Kim S.H., Lee H., Lee S.K., Cha C. (2017). Dual ionic crosslinked interpenetrating network of alginatecellulose beads with enhanced mechanical properties for biocompatible encapsulation. Cellulose.

[60] Kahya N., Golcu A., Erim F.B. (2019). Barium ion cross-linked alginate-carboxymethyl cellulose composites for controlled release of anticancer drug methotrexate. J. Drug Deliv. Sci. Technol..

[61] Swamy B.Y., Yun Y.-S. (2015). In vitro release of metformin from iron (III) cross-linked alginate-carboxymethyl cellulose hydrogel beads. Int. J. Biol. Macromol..

[62] Bulut E., Şanlı O. (2016). Novel ionically crosslinked acrylamide-grafted poly(vinyl alcohol)/sodium alginate/sodium carboxymethyl cellulose pH-sensitive microspheres for delivery of Alzheimer’s drug donepezil hydrochloride: Preparation and optimization of release conditions. Artif. Cells Nanomed. Biotechnol..

[63] St Dollente Mesias V., Penaloza D.P. (2021). Synthesis, Characterization, and Controlled Release Property Evaluation of Carboxymethyl Cellulose/Alginate(CMC/Alg) Encapsulated NPK Fertilizers. Philipp. J. Sci..

[64] Barbu A., Neamtu B., Zăhan M., Iancu G.M., Bacila C., Mireşan V. (2021). Current Trends in Advanced Alginate-Based Wound Dressings for Chronic Wounds. J. Pers. Med..

[65] Rego A. ALGS6 Ag AlginateWound Dressing & Aquacel Ag extra Hydrofiber Dressing with Silver and Strengthening Fiber 2017. https://www.accessdata.fda.gov/cdrh_docs/pdf17/K172570.pdf.

[66] Boateng J.S., Matthews K.H., Stevens H.N.E., Eccleston G.M. (2008). Wound healing dressings and drug delivery systems: A review. J. Pharm. Sci..

[67] Caló E., Khutoryanskiy V.V. (2015). Biomedical applications of hydrogels: A review of patents and commercial products. Eur. Polym. J..

[68] Clark R., Bradbury S. (2010). Silvercel^®^ Non-Adherent made easy. Wounds Int..

[69] Kammerlander G., Afarideh R., Baumgartner A., Berger M., Fischelmayer K., Hirschberger G., Hangler W., Huber A., Kramml M., Locherer E. (2008). Silvercel: Level two—Case studies. Clinical experiences of using a silver hydroalginate dressing in Austria, Switzerland & Germany. J. Wound Care.

[70] Edwards J. (2013). Use of Silvercel^®^ Non-adherent on burn wounds: A case series. Wounds UK.

[71] Silvercel Guidelines. http://www.woundsinternational.com/uploads/resources/d426e9e2f7575b30a7a42c5f5bbdb82a.pdf.

[72] Gray D. (2009). Silvercel^TM^ non-adherent dressing: Taking the pain out of antimicrobial use. Wounds UK.

[73] SMTL Dressings Datacard. http://www.dressings.org/Dressings/comfeel-plus.html.

[74] Ogushi Y., Sakai S., Kawakami K. (2007). Synthesis of enzymatically-gellable carboxymethylcellulose for biomedical applications. J. Biosci. Bioeng..

[75] Sarei F., Dounighi N.M., Zolfagharian H., Khaki P., Bidhendi S.M. (2013). Alginate Nanoparticles as a Promising Adjuvant and Vaccine Delivery System. Indian J. Pharm Sci..

